# Revisiting the Role of Leukocytes in Cystic Fibrosis

**DOI:** 10.3390/cells10123380

**Published:** 2021-12-01

**Authors:** Monica Averna, Paola Melotti, Claudio Sorio

**Affiliations:** 1 Department of Experimental Medicine (DIMES), University of Genova, 16132 Genova, Italy; monica.averna@unige.it; 2 Cystic Fibrosis Centre, Azienda Ospedaliera Universitaria Integrata Verona, 37126 Verona, Italy; paola.melotti@aovr.veneto.it; 3 Department of Medicine, General Pathology Division, University of Verona, 37134 Verona, Italy

**Keywords:** cystic fibrosis, leukocytes, inflammation, leukocyte subsets

## Abstract

Cystic fibrosis in characterized by pulmonary bacterial colonization and hyperinflammation. Lymphocytes, monocytes/macrophages, neutrophils, and dendritic cells of patients with CF express functional CFTR and are directly affected by altered CFTR expression/function, impairing their ability to resolve infections and inflammation. However, the mechanism behind and the contribution of leukocytes in the pathogenesis of CF are still poorly characterized. The recent clinical introduction of specific CFTR modulators added an important tool not only for the clinical management of the disease but also to the investigation of the pathophysiological mechanisms related to CFTR dysfunction and dysregulated immunity. These drugs treat the basic defect in cystic fibrosis (CF) by increasing CFTR function with improvement of lung function and quality of life, and may improve clinical outcomes also by correcting the dysregulated immune function that characterizes CF. Measure of CFTR function, protein expression profiling and several omics methods were used to identify molecular changes in freshly isolated leukocytes of CF patients, highlighting two roles of leukocytes in CF: one more generally related to the mechanism(s) causing immune dysregulation in CF and unresolved inflammation, and another more applicative role, which identifies in myeloid cells, an important tool predictive of the therapeutic response of CF patients. In this review we will summarize available data on CFTR expression and function in leukocyte populations and will discuss potential clinical applications based on available data.

## 1. Introduction

Cystic fibrosis (CF) is a severe autosomal recessive disease caused by variants in the cystic fibrosis transmembrane conductance regulator (CFTR) gene, which encodes a chloride ion channel. Dysfunction in CFTR leads, among others, to endobronchial infection, exaggerated pulmonary inflammation and airway obstruction that results in progressive lung disease. CF lung disease is characterized by chronic bacterial colonization, inflammation and an exaggerated neutrophilic inflammation, which is a recognized hallmark of the disease [1]. The identification of functional CFTR expression in myeloid cells reported in the last ten years or so shifted the perspective from a rather neutrophil- and epithelium-centric vision of CF disease [2] to a wider scenario. This scenario appears closer to the clinical observations that consider the interplay between multiple components of host defense, the airway inflammatory response and the multisystemic features of the disease, with inflammation playing a central role in its pathogenesis and evolution. Certainly, discussing inflammation nowadays means attempting to integrate the roles played by various leukocyte populations, a difficult task linked to a still-incomplete definition of the roles and the interplay between cells and mediators. In this review, we will discuss the currently available data linking the CFTR defect and its consequences with various leukocyte populations, namely myeloid and lymphoid cells, along with the possible relevance of these discoveries in our understanding of the disease and possible clinical applications.

## 2. Leukocytes and Cystic Fibrosis

### 2.1. Granulocytes

Neutrophils are the predominant immune cells infiltrating the airway mucosa and filling the intralumenal space of bronchioles in CF patients. However, they are unable to resolve CF bacterial infection [1]. The confirmation of CFTR expression on neutrophils has led to speculation that immune cell dysfunction may be implicated in CF lung inflammation [3,4]. Impaired degranulation appears to be a major cause of defective bacterial killing, as demonstrated by the normalization of this parameter upon pharmacological correction of the defect following treatment with modulators [5]. Phenotypic analysis revealed two distinct neutrophil populations in the CF airways; namely the CD63^lo^CD16^hi^ A1 and CD63^hi^CD16^lo^ A2 subsets [6], based primarily on low and high levels of NE-rich primary granule exocytosis, respectively [7,8]. A contribution by polymorphonucleate granulocytes to elevated gamma-glutamyltransferase (GGT) has been reported in the cystic fibrosis sputum [9]. GGT abundance in the lungs of CF patients was reported to be likely, depending on disease stage. The levels of this enzyme factor secreted by activated neutrophils in CF airways, associated with inflammation, should carefully be taken into account when evaluating the effects of GSH inhalation treatments [10] and, according to the results of a clinical trial, CF patients who do not benefit from inhaled glutathione might be identified by increasing sputum levels of GGT [11]. Neutrophils and bacteria colonizing CF airways, such as *P. Aeruginosa*, are sources of proteases [12] that were correlated with the development of bronchiectasis and the severity of lung damage [13,14]. Although clinical trials of neutrophil-focused therapies have been largely unsuccessful in CF, including inhaled NE inhibitors and recombinant human alpha-1 antitrypsin therapy [15,16] targeting NETs with nebulized DNase is a widely used, efficacious therapy that cleaves the DNA released from neutrophils, reduces mucous viscosity and improves lung function [17]. Neutrophils from CF patients with severe CFTR variants (e.g., F508del and G551D) have prolonged neutrophil survival and decreased phagocytosis and degranulation [18]. The introduction of CFTR modulator therapy permits us to study their effect on leukocyte populations not only ex vivo, but also in an in vivo setting, thus overcoming some criticism related to the effect of the lengthy isolation and culture conditions that might alter cells’ phenotypes and their consequent responses. The results of these studies were first reported for ivacaftor-treated cells and patients, and resulted in a clear effect on specific activation markers: in vivo and ex vivo exposure of G551D CF leukocytes to ivacaftor resulted in an altered activation profile, suggesting a mutation-specific leukocyte modulation [19]. Treatment of patients with G551D mutations with ivacaftor also has significant mutation-specific effects on myeloid cells [19]. Ivacaftor treatment induced beneficial changes in the markers of activation and adhesion consistent with neutrophils becoming less activated, and these observations were associated, in some cases, with significant changes in sweat chloride and lung function. The authors also suggest that the apoptosis defect they previously saw in CF neutrophils is dependent on severe CFTR dysfunction [20].

### 2.2. Monocytes/Macrophages

Di et al. [21] were the first to report a specific alteration in CF macrophages, showing that CFTR is expressed in the phagolysosomes of macrophages and has a critical role in phagosome acidification, a fundamental function for efficient bactericidal activity. These data were challenged in J774 and murine models where the authors failed to detect a difference in pH [22]. Another study confirmed no differences in lysosomal acidification between either uninfected (CFTR)-defective macrophages or normal macrophages treated with a CFTR-specific drug inhibitor. However, after ingestion of *B. cenocepacia*, acidification and phagolysosomal fusion of the bacteria-containing vacuoles occurred in a lower percentage of CFTR-negative macrophages than CFTR-positive cells [23]. Interestingly, Painter et al. reported defective intraphagolysosomal HOCl production in granulocytes from CF patients [24] and reported three mechanisms mediating the neutrophil-mediated killing of Pseudomonas Aeruginosa: (1) CFTR-dependent and oxidant-dependent, (2) chloride-dependent but not CFTR- and oxidant-dependent, and (3) independent of any of the tested factors [25]. Thus, they suggested that the alteration described in mono-macrophages might be subtle; under specific circumstances, alternative mechanisms might contribute to the intracellular killing defect identified in several of the studies mentioned below. Recently CRISPR/Cas9 deletion of the CFTR gene in human monocyte-derived macrophages has indicated that CF macrophages are unable to effectively phagocyte bacteria, and they are subsequently unable to mount effective intracellular killing responses. The authors suggested the cause was a mechanism linked to a reduced nicotinamide adenine dinucleotide phosphate (NADPH) oxidase assembly. At variance with most studies performed in primary human macrophages, it was notable that CFTR KO was associated with decreased production of several inflammatory cytokines in response to infection, suggesting the need to deepen our understanding of the role of CFTR and the influence of the microenvironment in macrophage reprogramming [26]. Another alteration that might influence innate response was reported by Wright et al., studying the role of scavenger receptors present in macrophages in CF. These authors reported a failure to express MARCO and mannose receptors in small sputum macrophages in CF. As MARCO mediates the phagocytosis of unopsonized particles, this finding suggests an impaired clearance of inhaled particles with increased inflammation and damage to the CF lung [27,28]. Another step forward was provided by the work of Bruscia et al., who, using a mice model of CF disease, demonstrated an increased inflammatory response in CF animals in comparison to WT mice, with an increased number of neutrophils and proinflammatory cytokines detected in their bronchoalveolar lavage fluid after LPS exposure [29]. Interestingly, the same authors show, by means of hemopoietic cell transplant, that CFTR^+/+^ hematopoietic cells, including macrophages, significantly dampen the proinflammatory response to LPS in CFTR^−/−^ animals. Along this line, Bonfield et al., showed that the absence of Cftr in myeloid-derived cells slows the resolution of inflammation and infection [30]. Indeed, in young children with CF and no active infection, inflammation can be detected in bronchoalveolar lavage (BAL) fluid, a process that was shown to be mainly driven via NLRP3-inflammasome activation [31,32]. Studies in primary macrophages indicate the presence, in the serum and monocytes of patients with CF, of an enhanced NLRP3-inflammasome signature with increased IL-18, IL-1b, caspase-1 activity and ASC speck release [33]. In a further study, the same group reported on how CFTR modulators downregulate this exaggerated proinflammatory response following LPS/ATP stimulation. Following in vitro exposure of CF monocytes to ivacaftor/lumacaftor or ivacaftor/tezacaftor, a significant reduction in IL-18 was reported, whereas IL-1b was only reduced with ivacaftor/tezacaftor. The data were expanded by in vivo study assessed over three months in 13 adults starting ivacaftor/lumacaftor and eight starting ivacaftor/tezacaftor. The results indicate a different response to the treatment with reduction of serum IL-18 and TNF, but IL-1b only declined following ivacaftor/tezacaftor. Interestingly, (LPS/ATP-stimulated) PBMCs, IL-18/TNF/caspase-1 were all significantly decreased, and IL-10 was increased with both combinations. Ivacaftor/tezacaftor alone showed a significant reduction in IL-1b and pro-IL-1b mRNA, thus indicating a different response to CFTR modulators and highlighting the complexity of the response in the patients, requiring the consideration of both the individual genotype and the response to the drug/drugs combination. Another study performed RNA-Seq in 20 patients with CF pre- and 6 months post-lumacaftor/ivacaftor initiation, and 20 non-CF healthy controls. The transcriptomic profile revealed marked overexpression of inflammation-related and apoptosis genes and significant under-expression of T and NK cell-related genes compared to the non-CF controls, which were decreased in CF cells following treatment with lumacaftor/ivacaftor, alongside the normalization of intracellular calcium levels [34]. Overall, these data indicate that CFTR modulators have potent innate anti-inflammatory properties, indicating IL-18 and IL-1b are potential reliable biomarkers for drug effectiveness at downregulating inflammation in CF [35]. Another class of inflammatory mediators is represented by the endogenous lipid mediator resolvin (Rv) D1. This is a potent regulator of resolution, a member of the family of pro-resolving lipid mediators (SPMs) produced mainly by macrophages and neutrophils via separate pathways from omega-3 polyunsaturated fatty acids (PUFAs) eicosapentaenoic acid (EPA) and docosahexaenoic acid (DHA). The role of RvD1 in CF was recently investigated and found to reduce chronic P. aeruginosa lung infection, inflammation, and damage-promoting resolution in vivo in CF mice. The mechanism was related to enhanced P. aeruginosa phagocytosis and reduced genes and proteins associated to NF-κB activation and leukocyte infiltration. Concentration of RvD1 in sputum from patients with CF was also inversely correlated to those of the cytokines and chemokines involved in CF lung pathology, suggesting an important role of this mediator in the pathogenesis of lung disease in CF, as well as a potential therapeutic application [36,37]. Recently, a specific defect altering agonist-dependent integrin activation (corrected in vitro by lumacaftor) was described in CF monocytes, leading to the proposal of the classification of CF as a new type of leukocyte adhesion deficiency (LAD) disease named LAD IV [38,39], adding further strength to the notion that an overall alteration in monocyte function, recruitment and activation is dysregulated in CF. Altogether, these data suggest that part of the CF phenotype expressed in humans might depend on the function of myeloid cells and can therefore represent a relevant target for current therapeutic strategies where the anti-inflammatory properties of CFTR modulator combinations, in addition to their ability to stimulate CFTR function, might contribute to improved clinical outcomes. The notion is further supported by studies in model animals where specific defects were recorded at birth, suggesting an intrinsic alteration to cell biology when CFTR function is altered. Indeed, studies on CFTR deficient pigs have shown altered responses of monocytes to pathogens tested on Pseudomonas aeruginosa infections [40], are in agreement with other modeling systems demonstrating a difference between the CFTR deficient environment and the healthy, non-CF environment [41,42,43,44]. These data not only underline the clinical relevance of these cells, but also suggest that probing CFTR expression/activity and associated biomarkers such as metalloproteinase 9 (MMP9) in peripheral blood leukocytes might have a possible clinical application such as the monitoring the efficacy of CFTR modulators [45,46,47,48].

### 2.3. Lymphoid Cells

Early studies identified CFTR expression and function in T cells [49]. Airway disease is present early in life in CF; even in asymptomatic infants, neutrophils accumulate in the airway lumen, while lymphocytes were found accumulated in the bronchial mucosa of CF children, with an increase of T cells during exacerbation [50]. Investigations of T cell–mediated responses support the concept that CF is predominately a Th2-mediated disease with high IL-13 and IL-4 release leading to aberrant immune responses [51], but involvement of Th17 cells also emerged. These cells produce IL-17A, a proinflammatory cytokine involved in the granulopoiesis, recruitment, activation and migration of neutrophils into sites of inflammation, inducing a cascade of other proinflammatory cytokines such as IL-6, IL-8, and granulocyte–macrophage colony-stimulating factor. Th1, Th2 and Th17-related cytokines were significantly up-regulated in the airways of patients with CF. This profile was associated with a potential risk factor for Pseudomonas aeruginosa infection [52]. T-regulatory cells (Tregs) seem to be involved in CF phenotypes, as suggested by their decreased numbers in *Cftr*^−/−^ mice linked to indoleamine 2,3-dioxygenase (IDO) deficiency, responsive to kynurenines treatment [53]. This alteration was confirmed in humans, where a quantitative and qualitative reduction of Tregs was detected. Their number was further decreased in the presence of a concomitant P. aeruginosa infection [54]. Furthermore, while no differences were detected in the peripheral blood Th17% between controls and patients with CF, high Th17% was strongly associated with poor lung function within the CF population. This supports its role as a contributing factor to lung damage, even if it still unclear whether this associates with the presence of lung damage secondary to infection or if it predisposes to it. The authors acknowledge that further studies are required to confirm whether Th17% in blood has any predictive value for lung function decline. Other regulatory T cells (FOXP3+, IL-10+, and TGF-β+CD4+) were elevated in the CF group compared to controls, with CD4+TGFβ+% possibly specifically associated with chronic P. aeruginosa infection [55]. These data also require validation in a larger cohort of patients to reduce confounding factors such as the presence and/or extent of chronic or, potentially, clinical laboratory “silent” infections that might introduce a bias when a highly dynamic population, such as that of leukocytes, is measured in patients. Another type of T cell was recently described having a potential role in CF: invariant NKT (iNKT) cells are a unique subset of T cells that express an invariant T cell receptor chain (Vα14-Jα18 segments in mice and Vα24-Jα18 segments in humans) recognizing glycolipid antigens in the context of CD1d and possibly involved in the control of autoreactive B cells triggered by cell death. Interestingly, their genetic deletion in CF mice prevents lung inflammation, suggesting a role for these cell types through the suppression of the auto-immune response induced by ceramide-mediated death of epithelial cells in CF lungs while attracting macrophages and neutrophils to CF lungs, resulting in chronic pulmonary inflammation [56].

Another relevant component of the immune response is natural killer (NK) cells. Recently, Mulcahy et al. reported significant correlations between proportions of NK and monocyte cell subsets and the clinical markers of lung function in CF patients, with an overall reduction of NK cells in CF patients compared with healthy controls, and an increase in both classical (CD16lo/negCD14hi) and proinflammatory (CD16hi CD14lo) monocytes, but no change in dendritic cells (DCs) in CF patients, compared with controls [57].

This said, some concerns were raised following the observation of an aberrant response against the CFTR antigen with evidence of the presence of T cells against human CFTR protein that are elicited in CF gene knockout (KO), heterozygous (Het), and wild-type (wt) mice, warning against potential autoimmune reactions following gene transfer attempts [58]. This risk might be also present following treatment with CFTR modulator therapy, which is expected to increase the expression of the CFTR protein at the cell surface, where it can be sensed by autoreactive T cells. Indeed. Calcedo et al. found non-CF subjects with low levels of self-reactive CFTR-specific T cells in the United States, and several CF patients with low to high levels of self-reactive CFTR-specific T cells in both the United States and the United Kingdom [59]. However, to our best knowledge, no adverse reactions of this type have been reported to date, despite the wide use of CFTR modulators in clinics.

Last but not least, although the role of CFTR in B cell activation and disease pathogenesis is still unclear, recent studies indicate its involvement in the regulation of B cell activation and lymphoid follicle development [60].

Overall, the role of NK and T cell subsets in CF seems to be established, while data are still scarce for B cells and still unfocused regarding a specific role. Clearly, more studies in the field are warranted to clarify these emerging issues and to build a more comprehensive picture of the altered balance of cells and mediators of immune response and inflammation so central in the pathogenesis of CF disease.

### 2.4. Platelets

Various reports indicate how platelets (PLTs), in addition to their well-appreciated hemostatic functions, play roles in inflammation and its resolution [61]. CF patients had increased circulating leukocyte-PLT aggregates and increased platelet responsiveness to agonists compared with healthy controls. Nevertheless, the authors could not identify CFTR expression and concluded that platelet activation in CF is the result of both plasma factor(s) and an intrinsic platelet mechanism via cyclic adenosine monophosphate (cAMP)/adenylate cyclase, but not via platelet CFTR [62]. However, more recent studies challenged this view, as CFTR expression was identified in human platelets by multiple assays (flow cytometry, Western blotting, immunohistochemistry, and immunogold electron microscopy). These platelets were found defective in their capability to produce lipoxins and arachidonic acid (AA) metabolites with potent anti-inflammatory and proresolution properties [63]. Animal models also support a proinflammatory role of the CF genotype: mice with global CFTR deletion have strikingly increased neutrophilic inflammation, NET formation, lung barrier disruption, and impaired bacterial clearance. Furthermore, these mice have increased platelet activation, which is also observed in human CF disease [64]. Moreover, platelet-specific deletion of CFTR in murine models of acute lung inflammation produced exaggerated, acute lung inflammation and platelet activation after intratracheal LPS or Pseudomonas aeruginosa challenge. CF subjects receiving CFTR modulator therapy showed partial restoration of CFTR function in the platelets, suggesting primary defects in CF platelets as an upstream trigger for neutrophilic inflammation and NET formation in CF lung disease [65].

## 3. Toward Clinical Applications

The confirmation of the expression of CFTR in non-epithelial cells and the significance of inflammation in the progression of CF lung disease have raised interest in the role of immune cells in CF. Particularly, polymorphonuclear neutrophils (PMNs) and monocyte/macrophage dysfunction [66] contributing to the disease pathogenesis [67] could represent intriguing cellular models to exploit, not only to better define the pathogenesis of the disease, but also to explore possible clinical applications. Various studies indicated that the restoration of CFTR function by correctors and/or potentiators in leukocytes promotes the normalization of inflammation and infection in CF patients, suggesting the potential of leukocyte outcomes as useful parameters to be examined in clinical trials utilizing CFTR modulators [19,35]. Considering that the clinical responses to CFTR modulators vary by, and even within, genotypes [68,69,70,71], and also that studies examining individuals before and after initiation of CFTR modulators have shown that these molecules do not reverse all disease manifestations [66,67,72], it is of great relevance to find biomarkers for monitoring therapies, particularly those predictive of an individual patient’s response. In the last decade, many studies have indicated that leukocytes, being easily and quickly isolated from patients, can be non-epithelial cellular models useful to monitor the responses of CF patients to the treatment during clinical trials and also as potential predicting biomarkers of the efficacy of therapies [45,47,73]. To this end, several approaches have been adopted. Recently, Guerra et al. demonstrated that CF human peripheral blood monocytes (MNCs) treated with ivacaftor rescued CFTR-dependent chloride efflux [73]. Moreover, since this MNC chloride efflux correlated with FVC and FEV1, they suggested that this test could be a surrogate marker similar to the respiratory function parameters. The same research group confirmed the hypothesis that CFTR-dependent chloride efflux in MNCs could be investigated as a sensitive outcome measure of therapy efficacy in CF patients. However, since these results were derived from a small cohort of patients, conducting studies in a higher number of patients is necessary. Other researchers selected systematic approaches, investigating changes in the proteomes or transcriptomes of peripheral blood monocytes from patients starting therapy with the CFTR potentiator ivacaftor. Hisert et al. identified post-ivacaftor changes in the monocyte plasma membrane proteome consistent with decreased interferon (IFN)-γ-related responses [74,75]. These studies highlight that ivacaftor alters the monocyte plasma membrane (PM) proteome but does not change systemic inflammation, suggesting that new approaches to developing therapeutics for modulating inflammation in CF are needed. More recently, a combination of transcriptomic data and clinical measurements have been applied with the aim of predicting patient responsiveness to the therapies [74,76]. These findings revealed that multiple transcriptional programs, including the pathways associated with immunity and inflammation, are modified in circulating CF monocytes after ivacaftor treatment. Moreover, Pedrazzi et al. combined the two approaches together identifying changes in specific proteomic profiles related to restored CFTR activity in CF leukocytes after ex vivo treatment with ivacaftor [47]. The limitation of these studies is that the changes considered in these cells following initiation of CFTR modulators were generally evaluated at only one time point. Thus, analyses of changes at multiple time points should be performed. The eventual effects of inflammation on CFTR function assays in leukocytes is another issue that deserves further investigation. Although a general application in establishing the diagnosis of CF seems not to be superior to currently available methods based on well-established laboratory and clinical criteria, CFTR expression and functional assays on peripheral blood samples should be considered to support diagnosis in difficult cases and for complex diagnosis when sweat chloride and genetic analysis are both inconclusive. Several proof-of-principle studies have indicated the capability of CFTR function assays in discriminating healthy carriers from CF patients and healthy controls [48,77]. Indeed, in some patients with difficult diagnoses, CFTR function has been evaluated in blood cells, providing overlapping results with certified procedures in the different tissues recommended for these cases such as intestinal current measurement (ICM) and/or nasal potential difference (NPD), according to the contraindications/availability in individual patients, suggesting that monocyte cells appear to be suitable for the purpose [78,79,80]. Regarding applications for the general CF population, various variants of the blood tests as previously described might be suitable to monitor compliance to and efficacy of the therapy due to sample accessibility and the low invasiveness of the sample retrieval procedure, but this approach requires further refinement and more extensive tests on the field.

## 4. Conclusions

**Figure 1:**
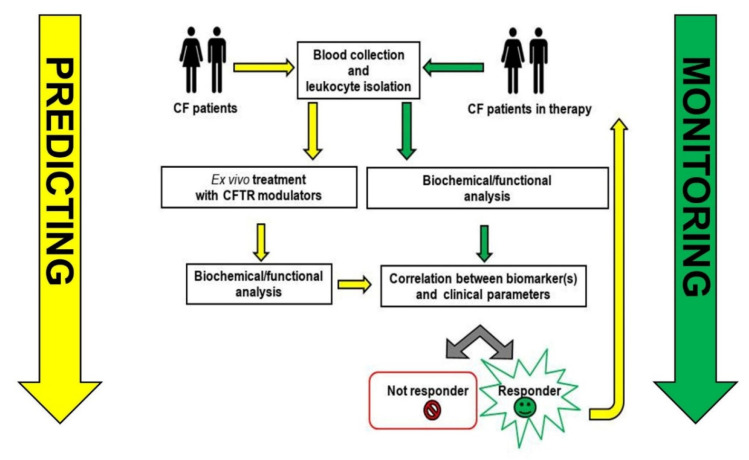
A possible application of leukocyte analysis in the context of the prediction and monitoring CF disease.

A bulk of the studies reveal that CFTR is expressed in leukocytes, with the most compelling evidence available for monocytes/macrophages, where biochemical, mRNA and electrophysiological data recorded in many independent laboratories converge to support the claim (Table 1). Adding CFTR assays in blood cells to NPD and ICM could, in single subjects, better reveal the functional impact of CFTR variants in different tissues, in particular for those with variable or unknown clinical consequences, with the advantages of the easy, quick, repeatable isolation of leukocytes from subjects of all ages, with minimal invasiveness and almost no clinical contraindications. These properties are not shared with the currently available certified tests in vivo (NPD) and ex vivo (ICM). Figure 1 summarizes possible applications of leukocyte analysis in the context of the prediction and monitoring of CF disease. In general, the bulk of the data discussed highlight the importance of strengthening studies on leukocytes, since their role in the complex pathophysiology of cystic fibrosis is so important. Although data to support the concept are available, additional studies are necessary to identify an easy and robust experimental approach that can lead to the identification of the best leukocyte biomarker(s) for predicting and monitoring the efficacy of the therapy most suitable for application in a clinical laboratory setting, in order to accelerate a much-needed personalized medicine approach in this field.

## Figures and Tables

**Table 1:** Summary of data supporting CFTR expression in leukocytes. FC: flow cytometry, WB: western blotting, IF: immunofluorescence; EM: electron microscopy; ND: not done.

	CFTR Function/Currents	CFTR Function/Others	Protein	mRNA	References
Mono/mac	Yes	Yes	FC/WB/IF	Yes	[21,46,48,77]
PMN	ND	ND	FC/WB/IF	Yes	[4,24,81]
B cells	Yes	ND	FC	Yes	[49]
T cells	ND	ND	FC	Yes	[49]
platelets	ND	Yes	ICH/WB/EM	undetectable	[63,65]
